# Case report: Brachial plexopathy caused by malignant peripheral nerve sheath tumor and review of the literature

**DOI:** 10.3389/fneur.2023.1056341

**Published:** 2023-01-16

**Authors:** Mengjie Chen, Xiuli Li, Xinhong Feng

**Affiliations:** Department of Neurology, Beijing Tsinghua Changgung Hospital, School of Clinical Medicine, Tsinghua University, Beijing, China

**Keywords:** brachial plexopathy, malignant peripheral nerve sheath tumors, diagnosis, EMG, case report

## Abstract

Brachial plexopathy (BP) is easily misdiagnosed due to its complexity and varying clinical presentation. Malignant peripheral nerve sheath tumors (MPNST) can accumulate in the brachial plexus and share symptoms with BP, which may hinder the differential diagnosis between BP induced by radiation or metastases, and MPNST-derived BP, in patients with a history of breast cancer and radiation exposure. A 34-year-old Chinese female presented with MPNST. The tumor involved the brachial plexus. She had a history of breast cancer and radiotherapy. The first consideration was radiation- or breast cancer metastasis-derived BP. Clinical examination was performed. Finally, a diagnosis of MPNST of the brachial plexus was made, which guided an accurate treatment plan. This report highlights the importance of correctly diagnosing BP etiology for guiding precise treatment. BP caused by MPNST needs to be considered in clinical practice, and biopsy plays a central role in the differential diagnosis. Complete local surgical resection can prolong survival of patients with MPNST and improve treatment prognosis.

## 1. Introduction

The brachial plexus provides motor and sensory innervation to the upper extremities. Brachial plexopathy (BP) is a peripheral neuropathy that occurs in the brachial plexus. The prevalence of cancer-associated plexopathy is ~0.4% in patients with cancer and 2−5% in those treated with radiotherapy, indicating that BP is a rare condition (1). Malignant peripheral nerve sheath tumors (MPNST) are derived from Schwann cells or generated through pluripotent cell differentiation of the neural crest. MPNST are rare and highly aggressive soft-tissue tumors. Type I neurofibromatosis is a major risk factor for MPNST (2, 3). However, ~45% of MPNST cases are incidental and 10% are due to previous radiation exposure (4, 5). MPNST can involve the brachial plexus and present with clinical features similar to those of BP, which may hinder the differential diagnosis between BP induced by radiation or metastases, and MPNST-derived BP, in patients with a previous history of breast cancer and radiation exposure. Improper treatment due to misdiagnosis can lead to medicolegal disputes and delays in effective treatment of malignant tumors, markedly affecting patient clinical outcome. Therefore, it is recommended that in patients with BP, particularly those with a history of breast cancer and radiotherapy, the diagnosis of the cause should be achieved as it helps determine the accurate treatment decision. A representative case is discussed in the following section.

## 2. Case presentation

A 34-year-old female patient presented with weakness that progressed over 1 year and progressively involved her entire left upper limb. She had needle-like intermittent pain in left upper limb with visual analog scale (VAS) score of 4-5 points, and attack duration of 1–2 s occurring several times a day but mostly at night. Six months ago, the above symptoms worsened. VAS score was 6–7 points, and there was increased pain frequency. She was unable to lie on her back. Three weeks prior, she could not lift her left arm, and the pain radiating from the left shoulder blade to the fingers worsened. The patient had a history of breast cancer and had undergone standard radiotherapy and chemotherapy. No other pertinent history was noted.

**Figure 1:**
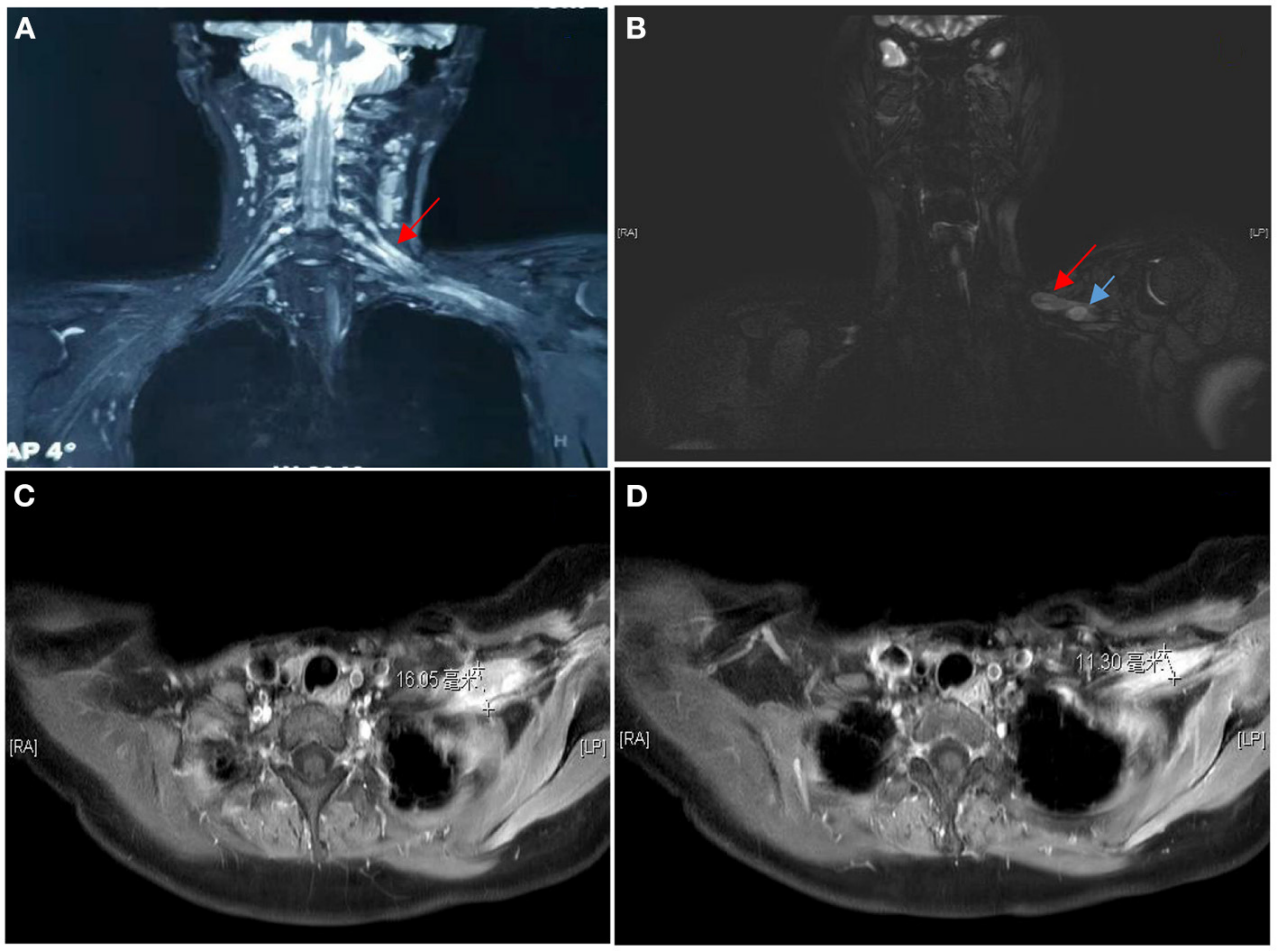
MRI of the brachial plexus showed the C5/C6 roots, upper and middle trunks and all strands of the left brachial plexus were significantly swollen and thickened, with T2W high signal, the right brachial plexus had good continuity, with no clear thickening and no signal enhancement **(A)**. MRI showed multiple nodules within the brachial plexus in the left subclavian region **(B)**, the two that could be distinguished were ~16 mm **(C)** and 11 mm **(D)** in diameter respectively.

**Figure 2:**
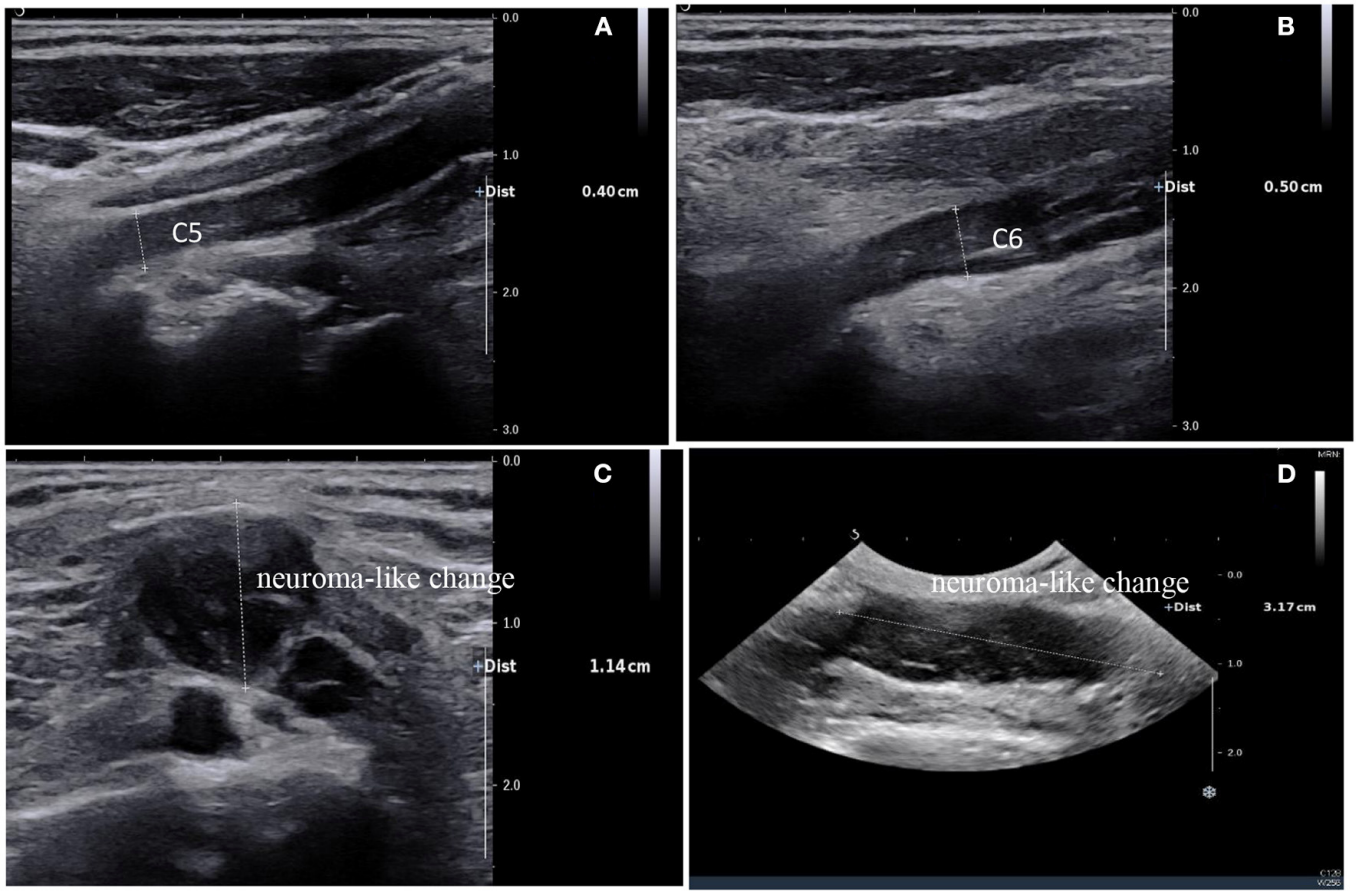
Ultrasound of the brachial plexus showed a high degree of thickening of the C5 and C6 nerve roots, ~0.4 cm and 0.5 cm respectively **(A, B)**; diffuse edema of the superior, middle, and inferior trunks were seen in the interosseous sulcus; Brachial plexus neuroma-like change of brachial plexus in the supraclavicular fossa, ~1.14 cm thick at most and 3.17 cm long **(C, D)**.

**Figure 3:**
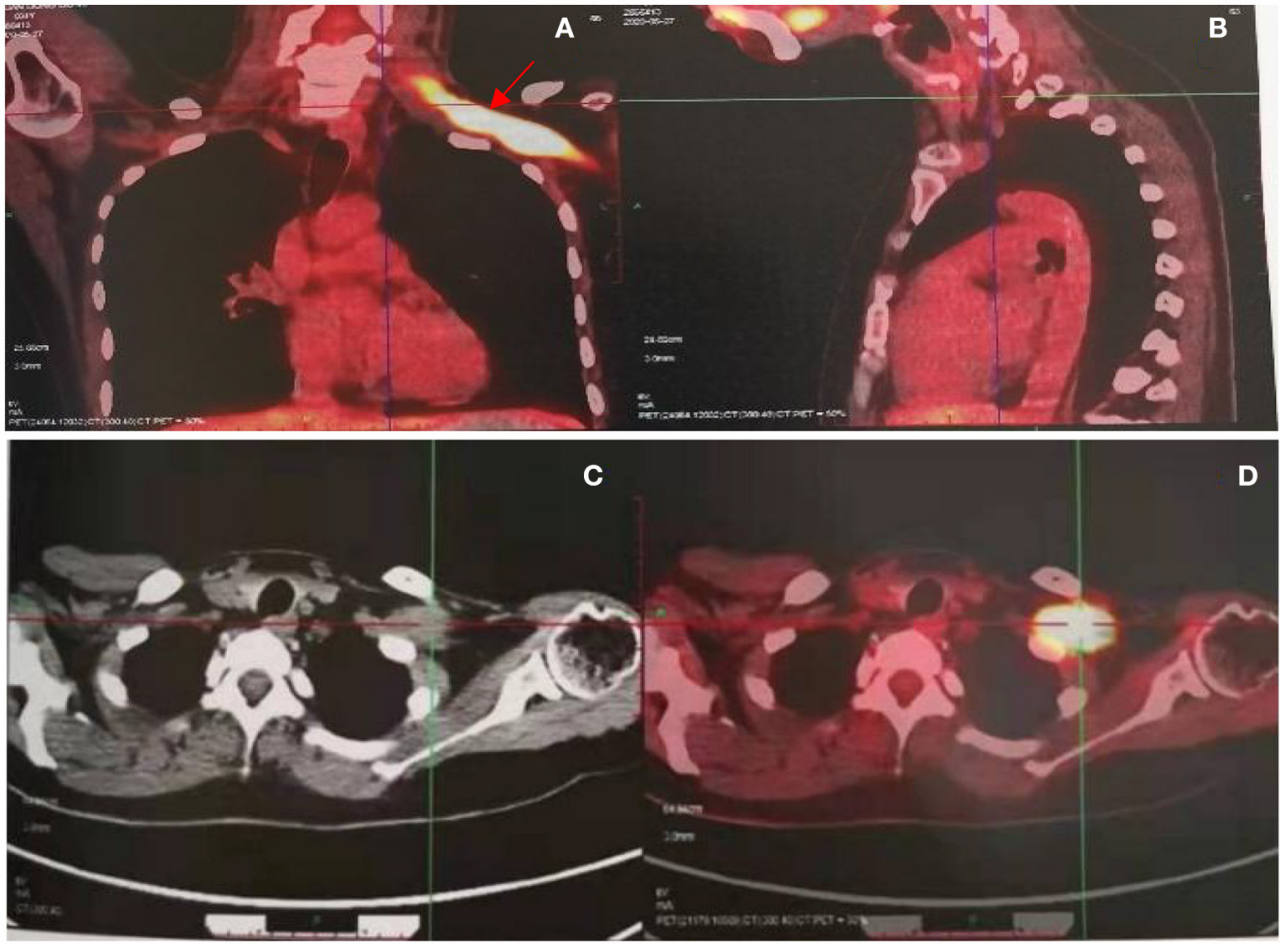
PET CT revealed increased radioactive uptake in the left brachial plexus (SUV max 24.4) **(A, B)**, involving a length of 9.3 cm **(C, D)**.

**Figure 4:**
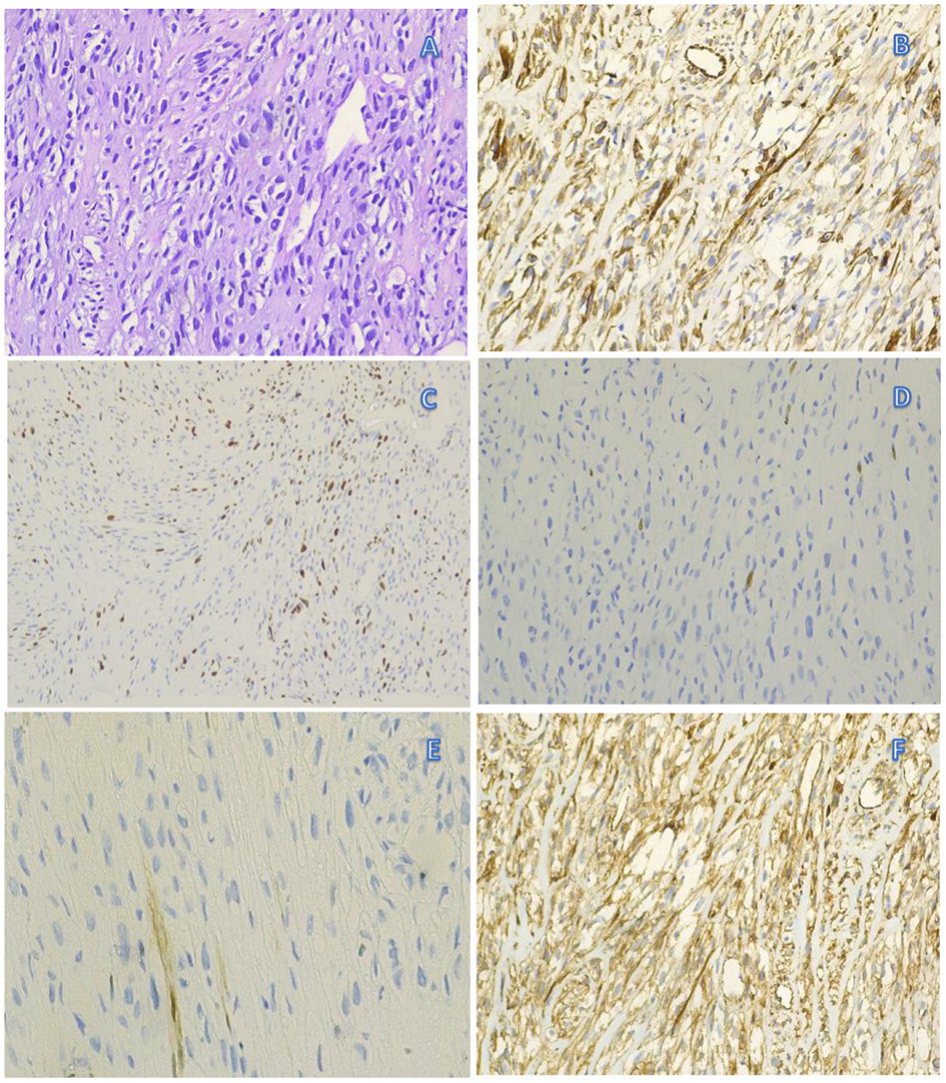
Pathology (tissue of brachial plexus swelling in the left supraclavicular fossa): diffuse lamellar arrangement of the spindle or oval cells, mild-moderate heterogeneity of the nuclei, nuclear schism 4/10HPF, some interstitial vitreous changes, chondrogenic differentiation visible in focal areas **(A)**. Immunohistochemistry: Vimentin (+) **(B)**, Ki-67 (25%+) **(C)**, SOX10 (individual cells+) **(D)**, S-100 (individual cells weakly+) **(E)**, P120 (+) **(F)**. In summary, a diagnosis of malignant peripheral schwannoma was considered.

Clinical examination revealed decreased muscle strength and muscle atrophy in the left upper limb. Left biceps and radial reflexes were not elicited; the rest of the neurological examination was normal. Electrophysiological studies were performed to evaluate the brachial plexus. Nerve conduction studies showed that the motor and sensory nerves of the right upper limb were normal, whereas the left brachial plexus was injured with predominant axonal injury. In addition, needle electromyography revealed damage to the left brachial plexus. Laboratory examinations revealed that the blood levels of the tumor marker Squamous Cell Carcinoma Antigen were 2.1 ng/ml (reference value < 1.5 ng/ml). Further neuroimaging was performed. Magnetic resonance imaging (MRI) of the brachial plexus revealed thickening and edema of the left brachial plexus (Figure 1). Ultrasound of the left brachial plexus showed significant edema and thickening of the left brachial plexus roots, trunk, and strands, mostly pronounced in the supraclavicular fossa segment, with local thickening showing neuroma-like changes, ultrasound of the left upper arm nerve showed edema of some nerve bundles (Figure 2). The patient was a young woman with an insidious and progressive onset of symptoms, with pain in the left upper limb as the first symptom and progressive weakness affecting the entire upper limb function. She had a history of previous tumor treatment, the possibility of either radiation-induced BP or metastatic BP resulting from breast cancer was considered. Radiation-induced BP, with predominant neurofibromas on the nuclear magnetic structure, was less consistent with this patient's symptomatology. Further, positron emission tomography-computed tomography (PET-CT) revealed increased radioactive uptake in the left brachial plexus (SUV 24.4), indicating a tumor (Figure 3). Finally, we performed a pathological examination of tissue of brachial plexus swelling in the left supraclavicular fossa. The pathological pictures showed a diffuse laminar arrangement of spindle or oval cells with mild-moderate heterogeneity of the nuclei, along with some vitreous changes and chondrogenic differentiation. Morphology and positive immunohistochemical presentations (Vimentin, Ki-67, SOX10, S-100, P120) of nerve biopsy confirmed an MPNST (Figure 4).

Based on our follow-up with the patient, we noted that she had undergone complete local surgical resection at another hospital. After 2 months of post-operative rehabilitation, the patient's severe pain in the left upper limb was relieved and the muscle strength could resist partial resistance. The prognosis of the patient was good.

## 3. Discussion

The brachial plexus comprises the ventral nerves of the C5-T1 spinal nerves and innervates the upper limbs, shoulders, and upper chest. The brachial plexus can be localized based on neurological and neurophysiological examination (6, 7). BP is one of the most severe types of peripheral nerve injury because of its complicated anatomical structure (8). BP is divided into two main categories, traumatic and non-traumatic, depending on the clinical situation (9). Non-traumatic BP usually occurs because of idiopathic brachial neuritis, BP caused by direct tumor invasion or metastasis, radiation-related BP, thoracic outlet syndrome, and iatrogenic injury, all of which can cause partial or complete loss of upper limb function (10).

BP-associated with breast cancer can occur due to radiation injury or metastatic spread of the tumor (11, 12). In our case, the first consideration for diagnosis was the more commonly occurring radiation-induced brachial plexus neuropathy (RIBPN) (10, 13, 14). It should be noted that the differential diagnosis between neoplastic-derived BP and RIBPN is complex in clinical practice. In terms of clinical features, pain is usually more severe in neoplastic BP than that in RIBPN, affects the lower brachial plexus more often, and is more commonly associated with Horner's syndrome (14, 15). In addition, sensory abnormalities and muscle weakness are usually more severe in tumor-induced brachial plexopathies than in RIBPN (16). The presence of myofibrillation on neurophysiological examination supports the diagnosis of RIBPN, together with the presence of myofibrillatory discharges (17–21). An electromyogram (EMG) can detect ~30% of myofibrillatory discharges, which are usually undetectable in cases with tumor infiltration; however, myofibrillatory discharges are not a common feature of RIBPN. MRI is a non-invasive test commonly used to distinguish brachial plexus pathologies (22, 23) and has a higher resolution of anatomical structures compared to other imaging techniques such as ultrasound. Conventional MRI sequences used to evaluate the brachial plexus typically include T1-weighted (T1W) and fat-suppressed T2-weighted (T2W) images. Upon fibrosis within and around neurons due to radiotherapy, T2W images show an equal to low signal relative to that of the muscle. Conversely, T2W sequences show a massy high signal in the presence of brachial plexus tumor infiltration. In addition, nerves with radiological brachial plexus injury show mainly diffuse, homogeneous, and symmetrical swelling on MRI (24). Tumors usually appear on MRI as a heterogeneous, asymmetric enlargement of the lesion and as an enhancing mass (25, 26). Based on these parameters, it is possible to differentiate between radiological brachial plexus injury and a tumor to a certain extent, and diffusion-weighted imaging (DWI) sequences may be effective in differentiating benign or malignant peripheral nerve mass-like or infiltrative lesions (27). In our case, the nerve ultrasound and MRI findings were not consistent with brachial plexus injury after radiotherapy, particularly the nodular and tumor-like thickening of the brachial plexus on ultrasound, suggesting a tumor association (28, 29).

PET is important for identifying the tumor-associated brachial plexus as it can detect the hypermetabolic manifestations of tumors. RIBPN generally reflects a slight avidity. Increased radioactivity uptake in the left brachial plexus on PET suggests the presence of a tumor (26, 30). In conjunction with this case, elevated tumor markers suggest the possibility of tumor-induced BP. However, this patient was treated with a full course of radiotherapy and continued endocrine therapy after breast-conserving surgery, and no tumor recurrence was observed at regular follow-up. The diagnosis of MPNST as the etiology of BP was confirmed with biopsy. The pathological diagnosis of MPNST requires a combination of morphology, immunohistochemistry and clinical history. Under the microscope, MPNSTs are typically infiltrative lesions that show different cell morphologies (including spindle-shaped, epithelioid, pleomorphic, or oval-shaped). Spindle-shaped cells MPNST are often arranged in long fascicles. The nucleus is also diverse in morphology, displaying elongated, conical, curved or wavy shapes. Widespread heterogeneity of the nucleus and nuclear schism are common. Interstitial vitreous changes and chondrogenic differentiation can be found on pathological images (31). These diagnostic pathomorphological manifestations are consistent with our case. Immunohistochemically, as the Schwann differentiation of tumors is highly variable and incomplete, S-100 protein expression is usually more restricted, showing weak positivity (32). Nuclear expression of Sox10 is an essential neural crest transcription factor for specification and maturation of Schwann cells (33). The expression of Ki-67 protein is recognized to be closely related to the cell division cycle and can reflect the malignancy of the tumor (34). Vimentin protein is an important component of the cytoskeleton. Positive immunohistochemistry indicates that the tumor is likely to originate from mesenchymal tissue (35). P120 is involved in intercellular adhesion and signal transduction in several cell types and is associated with tumor formation (36). Immunohistochemistry is very valuable for the diagnosis of MPSNT.

Breast and lung cancer are the most common cancer types causing BP. Primary brachial plexus tumors (e.g., nerve sheath tumors and neurofibromas) are uncommon and mostly isolated. These tumors rarely cause symptomatic plexopathy (10, 37). However, multiple tumors occur in patients with type I neurofibromatosis, and these are more likely to present as pain with associated functional deficits. Both type I neurofibromatosis and malignant transformation of these tumors are rare. MPSNT accounts for 5–10% of all soft tissue sarcomas and is much less common when it involves the brachial plexus; 10% of MPSNT is due to previous radiation exposure (5, 38, 39). In the present case, a diagnosis of radiation-induced MPNST was considered in the context of the patient's past medical history as a relationship between radiotherapy and MPNST progression had been demonstrated. Radiotherapy causes chromosomal damage and induces abnormal and atypical cytological proliferation of Schwann cells. It accelerates the progression of malignant nerve sheath tumors, particularly in susceptible patients (40, 41). As peripheral nerves are very sensitive to radiotherapy, persistent inflammation after this treatment leads to changes in the peripheral nerve microenvironment and a higher proliferation rate. Radiotherapy can also cause lymphatic obstruction and fibrotic damage to the perineural vessels, allowing mutated Schwann cells to evade immune surveillance and continue to proliferate (42).

Complete local surgical resection can prolong the survival of patients with malignant peripheral schwannomas. For radiation-induced MPNST, the median survival time was 5 months for patients without surgery, 16 months for patients with complete surgical resection, 28.3% for 2-year survival, 12 months for patients with incomplete surgical resection, and 7.5% for 2-year survival. In general, radiation-induced MPNST is associated with poorer prognosis compared with that for sporadic MPNST (43). In these circumstances, EMG can detect abnormal changes in electrical activity that occur during surgery, which may reveal potential nerve damage. Thus, the use of intraoperative neurophysiological monitoring (IONM) for electrical activity ensures the best possible nerve integrity, avoids unnecessary damage, and improves treatment prognosis (44).

In conclusion, a complete diagnostic pathway for BP in a patient with a history of breast cancer and radiotherapy is presented here. This case report highlights the importance of correctly diagnosing the etiology of BP in order to guide precise treatment. MPNST-derived BP, together with RIBPN and BP derived from breast cancer metastases require attention in clinical practice in a patient population with a history of breast cancer and radiotherapy, with biopsy playing a key role in the differential diagnosis. Concerning treatments, complete local surgical resection can prolong the survival of patients with MPNST and improve treatment prognosis. Importantly, the risk of brachial plexus injury secondary to MPNST should be considered before patients are treated with radiotherapy for the primary tumor lesion, and long-term follow-up along with neurological examination of patients after radiotherapy is necessary. It is also pertinent to investigate the molecular mechanisms underlying radiation-induced MPNST.

## Data availability statement

The original contributions presented in the study are included in the article/supplementary material, further inquiries can be directed to the corresponding author.

## Ethics statement

The studies involving human participants were reviewed and approved by Ethics Committee of the Beijing Tsinghua Changgung Hospital, School of Clinical Medicine, Tsinghua University. The patients/participants provided their written informed consent to participate in this study. Written informed consent was obtained from the individual(s) for the publication of any potentially identifiable images or data included in this article.

## Author contributions

MC: drafting/revision of the manuscript for content, including medical writing for content, and analysis or interpretation of data. XL: study concept or design and analysis or interpretation of data. XF: drafting/revision of the manuscript for content, including medical writing for content, major role in the acquisition of data, and study concept or design. All authors contributed to the article and approved the submitted version.
